# ECO: An Integrated Gene Expression Omnibus for Mouse Endothelial Cells *In Vivo*

**DOI:** 10.3389/fgene.2022.844544

**Published:** 2022-03-04

**Authors:** Xiangyi Deng, Fan Yang, Lei Zhang, Jianhao Wang, Boxuan Liu, Wei Liang, Jiefu Tang, Yuan Xie, Liqun He

**Affiliations:** ^1^ Department of Neurosurgery, Tianjin Medical University General Hospital, Tianjin Neurological Institute, Key Laboratory of Post-Neuroinjury Neuro-Repair and Regeneration in Central Nervous System, Ministry of Education and Tianjin City, Tianjin, China; ^2^ Key Laboratory of Ministry of Education for Medicinal Plant Resource and Natural Pharmaceutical Chemistry, National Engineering Laboratory for Resource Developing of Endangered Chinese Crude Drugs in Northwest of China, College of Life Sciences, Shaanxi Normal University, Xi’an, China; ^3^ Precision Medicine Center, the Second People’s Hospital of Huaihua, Huaihua, China; ^4^ Trauma Center, First Affiliated Hospital of Hunan University of Medicine, Huaihua, China; ^5^ Department of Immunology, Genetics and Pathology, Uppsala University, Uppsala, Sweden

**Keywords:** endothelial cells, gene expression, RNAseq, database, integration

## Abstract

Endothelial cell (EC) plays critical roles in vascular physiological and pathological processes. With the development of high-throughput technologies, transcriptomics analysis of EC has increased dramatically and a large amount of informative data have been generated. The dynamic patterns of gene expression in ECs under various conditions were revealed. Unfortunately, due to the lack of bioinformatics infrastructures, reuse of these large-scale datasets is challenging for many scientists. Here, by systematic re-analyzing, integrating, and standardizing of 203 RNA sequencing samples from freshly isolated mouse ECs under 71 conditions, we constructed an integrated mouse EC gene expression omnibus (ECO). The ECO database enables one-click retrieval of endothelial expression profiles from different organs under different conditions including disease models, genetic modifications, and clinically relevant treatments *in vivo*. The EC expression profiles are visualized with user-friendly bar-plots. It also provides a convenient search tool for co-expressed genes. ECO facilitates endothelial research with an integrated tool and resource for transcriptome analysis. The ECO database is freely available at https://heomics.shinyapps.io/ecodb/.

## Introduction

Endothelial cells (ECs) are single-layered squamous cells distributed on the inner surface of the vasculature, constructing a barrier between the vasculature and tissues and controlling the exchange of substances and fluids (Krüger-Genge et al., 2019). ECs are involved in many essential physiological functions, such as regulating vasoconstriction and vasodilation, blood coagulation, paracrine action, angiogenesis, and constitute barriers (Reglero-Real et al., 2016; Wong et al., 2017; Paone et al., 2019). Dysfunction of EC is the driving factor for many diseases, including atherosclerosis, cancer, hypertension, glomerular disease, and inflammation (Goveia et al., 2014; Li et al., 2019). Uncovering the molecular mechanism of endothelial cells in these pathological conditions is essential to understand the occurrence and treatment of diseases.

With the rapid development of high-throughput sequencing technologies in the last decades, especially the wide use of RNA sequencing, the molecular level analysis of EC has increased significantly and a variety of EC transcriptomics datasets have been accumulated in the public domain (Khan et al., 2019; Munji et al., 2019). Their raw RNAseq data generated by high-throughput sequencing are deposited in the public databases, such as Gene Expression Omnibus (GEO) (Barrett et al., 2007) and ArrayExpress (Parkinson et al., 2007), but unfortunately, it is difficult for researchers without bioinformatics skills to process these raw data and extract the desired information. In some other fields, there are already some databases that provide practical functions to greatly promote the development of this field, such as the Allen Brain Atlas (Lein et al., 2007) for neuroscience and ONCOMINE (Rhodes et al., 2004) for oncology. For EC data, the effort of integrating has been initiated, for example, EndoDB, which has made a collection of EC data (Khan et al., 2019). However, there is still a lack of database integrating all latest RNAseq data and also providing user-friendly analysis functions and visualization tools.

Here, we integrated all freshly isolated EC bulk RNA sequencing data from public sequence databases, processed them with a standardized pipeline, and constructed a user-friendly online database, ECO. It provides a one-click search tool for *in vivo* EC profiles for each gene in various conditions including pathological alterations, genetic modifications, and other treatment conditions, in the form of easily understandable bar-plots. Also, the database provides a search function to find genes with similar expression profiles, which may generate interesting hypothesis for future research.

## Methods

### Retrieval of EC RNA Sequencing Datasets

We first conducted a systematic literature search for murine *in vivo* EC bulk RNAseq studies in PubMed, the NCBI GEO database, and the ArrayExpress database. It resulted in 19 RNA studies for EC under various conditions. They include 71 EC conditions. Each condition has multiple replicated samples, and in total, there are 203 samples. The raw sequence data for each condition, including the raw data for its exact control group, were obtained from the NCBI Short Read Archive (SRA) or ArrayExpress database.

### Data Preprocessing on Galaxy

The raw sequence data obtained from SRA and ArrayExpress were preprocessed with the Galaxy online server (Jalili et al., 2020) (https://usegalaxy.eu/, version: 20.09) using a standardized procedure for all datasets. The detailed procedure is described in the Galaxy RNA-seq analysis instruction (https://training.galaxyproject.org/training-material/topics/transcriptomics/tutorials/rna-seq-reads-to-counts/tutorial.html).

The sequence data were uploaded in two ways: for the data available in SRA, the SRA-tools (Leinonen et al., 2011) (version: 2.10.8) in Galaxy were used to upload these datasets reads in the FASTA/Q format from the NCBI; for the other datasets from ArrayExpress, the ArrayExpress FTP download links were used. The FASTQ sequence files were then aligned to the referenced mouse genome assembly (GRCm38/mm10) obtained from the UCSC Genome Browser database (Navarro Gonzalez et al., 2021) using the HISAT2 tool (Kim et al., 2015) (version: 2.1.0) on Galaxy. The gene annotation file GTF (2020, ncbiRefSeq, mm10) was also obtained from the UCSC Genome Browser database, which was consistent with the genome sequence file. The alignment bam files were then input to the featureCounts tool (Liao et al., 2014) (version: 2.0.1, with default parameters) to get the raw read counts for each genes (feature count files). In total, 203 samples were quantified and their count data were processed in R (version: 4.0.3) for downstream analysis.

### Data Normalization

In order to compare the EC expression level among different samples in different conditions, all the raw count data were normalized using rpkm function in the edgeR package (version: 3.32.0). The FPKM values for each sample were calculated, and then, the average expressions and standard deviations for each of the 32 conditions (71 bars in the FPKM plot) were calculated in R. The result for each gene was visualized in bar-plot using the ggplot2 package (version: 3.3.2).

### Differential Expression Gene Analysis

The gene expression raw count files were imported into the limma package (version: 3.46.0) in R, and the voom function was used to compare the gene expression between two groups (treated versus control) with the default parameters. To remove low-expression genes in each sample, the genes which were detected in only one sample were filtered out. To visualize the differential expression profiles among the 40 comparison groups, the fold changes and the standard deviations for each gene were visualized in bar-plots.

### Correlation Analysis

To search for the genes with similar expression profiles with a query gene, the corr.test function from psych package (version: 2.0.12) was applied. The correlation coefficient and the *p* values were calculated. The sorted result was stored in a table and is available for download through our ECO database. In addition, to better illustrate the correlation result, we chose the 10 most correlated genes to the query gene and generated a heatmap with the pheatmap package (version: 1.0.12).

### ECO Web Tool Construction

Our ECO database, an interactive web application, is built mainly using the R Shiny package (version: 1.6.0), as well as the other auxiliary packages including shinythemes (version: 1.2.0), ggplot2 (version: 3.3.3), and ggh4x (version: 0.1.2.1). The ECO database is available for free at https://heomics.shinyapps.io/ecodb/.

## Results

### Construction of ECO

**FIGURE 1:**
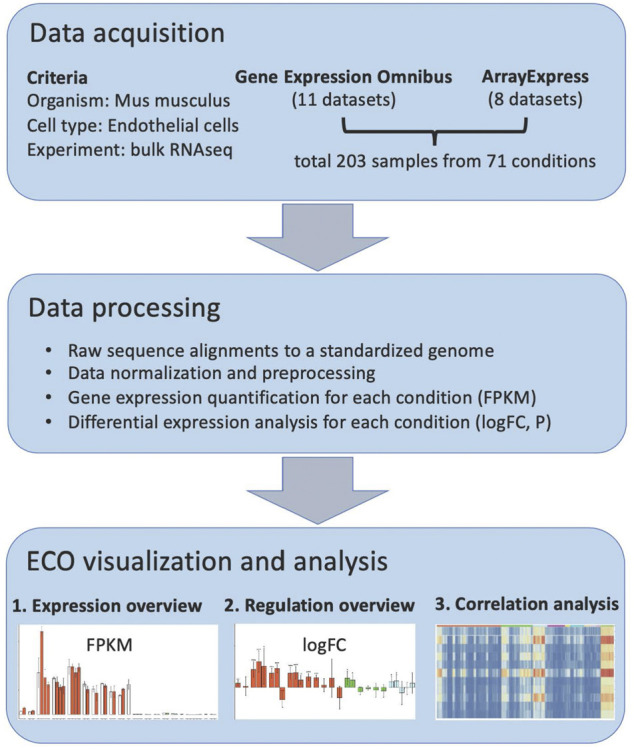
ECO workflow.

In order to construct a comprehensive omnibus of mouse *in vivo* EC RNAseq profiles, we performed literature mining and identified 19 currently available RNA studies (Supplementary Table S1), which cover EC in a variety of pathological alterations, genetic modifications, and other stimulated conditions. In these studies, freshly isolated ECs were analyzed with RNA sequencing. In total, there are 203 samples covering 71 *in vivo* conditions from 10 organs. These data composite the base for ECO, and they were processed as shown in the workflow (Figure 1). First, their raw data were obtained from the GEO database or ArrayExpress database, respectively. The sequence data were aligned to a standardized mouse genome assembly (GRCm38/mm10), and gene expression in each sample was quantified using the Galaxy analysis platform (Jalili et al., 2020). The gene expression levels in each condition were then summarized (average FPKM and standard deviation) and available for bar-plot visualization in the ECO database (https://heomics.shinyapps.io/ecodb). Also, the gene expression in each condition was compared with its respective control by differential expression analysis, and log scaled fold change (logFC) and *p* values were calculated, which are also illustrated with the bar-plot in the database. Besides the display of expression profiles in ECs, ECO can further identify the genes which showed similar expression profiles with the queried gene by using correlation analysis. The results were shown both as a heatmap and table.

### Investigation for Inter-Organ Heterogeneity of ECs by Using ECO

ECs in different tissues have heterogeneous phenotypes for their distinct physiological needs (Kalucka et al., 2020). For instance, brain ECs form tight junctions and express active transporters to restrict diffusion, known as the blood–brain barrier (BBB) (Daneman and Prat, 2015). In contrast, ECs in the kidney are associated with fenestrae to allow efficient passage of high-volume fluids and formation of urine (Dumas et al., 2021). EC profiles from 10 organs, including the brain, lung, bone, kidney aorta, liver, eye, muscles, lymph node, and embryo, were cataloged in ECO. Users can access and download the expression of the gene of their interest in ECs of different organs in ECO by simple one-click of FPKM button. Also, users can input a customized gene list to analyze their overall gene expression enrichment pattern in a heatmap.

**FIGURE 2:**
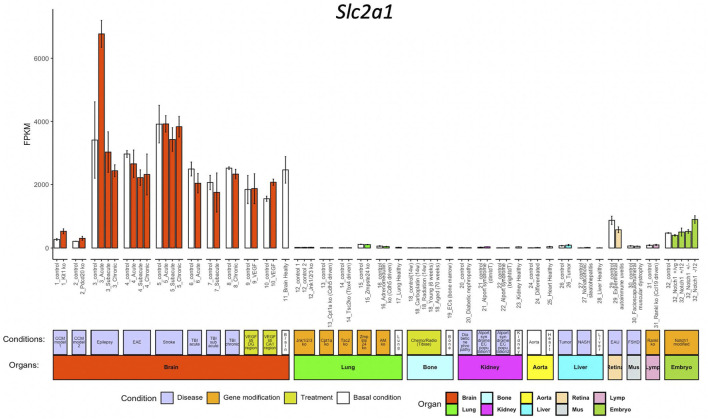
Bar-plot of *Slc2a1* expression in different conditions. The *x*-axis shows the 71 conditions, and the *y*-axis shows the normalized FPKM values. Each bar shows the average expression (+/− standard deviation) in each condition. The bars are colored according to their organ origins, and all basal/control conditions are shown with white color (high-resolution image is available in the ECO online database).

We use *Slc2a1* as an example to explore the inter-organ heterogeneity of a given gene. *Slc2a1*, encoding Glut1, which is highly expressed in BBB ECs but not peripheral ECs and facilitates glucose transport over BBB (Zheng et al., 2010). When we access *Slc2a1* expression by pressing the FPKM button after entering the gene symbol in the query interface, we get the normalized data bar-plot visualization for 32 sub-groups from 10 organs. As expected, *Slc2a1* is highly expressed in ECs from the brain, but almost absent in other organs (Figure 2).

### Exploring EC Gene Alterations in Response to Disease, Genetic Manipulations, or Other Stimulations *In Vivo* by Using ECO

**FIGURE 3:**
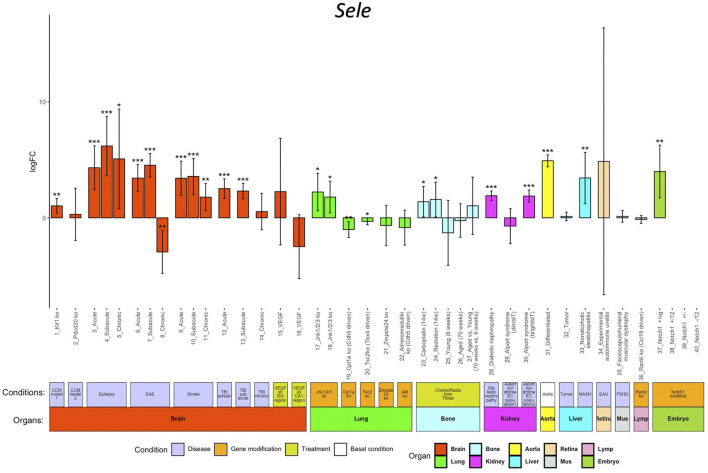
*Sele* regulation in different conditions. The *x*-axis shows the 40 conditions, and the *y*-axis shows the log2 scaled regulation fold change (logFC). Each bar shows the average fold change with confidence interval in each condition. The bars are colored according to their organ origins (high-resolution image is available in the ECO online database).

ECs participate in the regulation of multiple processes including angiogenesis, coagulation, and inflammation. Endothelial dysfunction is associated with many pathological alterations and aggravates progression of multiple life-threatening diseases including cancers, cardiovascular disease, diabetes mellitus, and renal disorders. In ECO, we collected EC transcriptomes from eleven mice disease models (cerebral cavernous malformation (CCM), epilepsy, experimental autoimmune encephalomyelitis (EAE), stroke, traumatic brain injury (TBI), diabetic nephropathy, Alport syndrome, liver cancer, non-alcoholic steatohepatitis (NASH), experimental autoimmune uveitis (EAU), and facioscapulohumeral muscular dystrophy (FSHD)), seven gene-modified animal models (*Jnk1/2/3* EC-specific deficient, *Cpt1a* EC-specific deficient, *Tsc2* mesenchyme cell-specific deficient, *Zmpste24* deficient, adrenomedullin (AD) EC-specific deficient, *Tankl* stroma cell-deficient, and EC-specific *Notch1* mutants), and two clinically relevant treatments (VEGF stimulations and chemo/radiotreatment) (Supplementary Table S1). The users can access the alteration of the genes of their interest in response to the abovementioned conditions compared to their control by clicking the logFC button. The result is illustrated in a bar-plot with 40 columns; each column represents the log2 scaled fold change, and its statistical significance (*p* value range) is indicated by asterisks (Figure 3).

We use *Sele* as an example to demonstrate the exploration of its regulation in different pathological conditions, genetic modifications, and treatments *in vivo*. E-selectin, encoded by *Sele*, is upregulated in ECs in response to pro-inflammatory signals, promoting the rolling and adherence of immune cells to ECs for their diapedesis (Jubeli et al., 2012). Inflammation is closely linked in the EC dysfunction in multiple diseases (Steyers and Miller, 2014). As shown in Figure 3, ECO provides a comprehensive portrait for *Sele* in different pathological conditions. *Sele* was upregulated in ECs from eight disease models including CCM, EAE, stroke, TBI, epilepsy, AOD, diabetic nephropathy, Alport syndrome, and NASH, highlighting the broad role of *Sele* in multiple disease progressions (Silva et al., 2017).

### Predicting the Function of Poorly Characterized Genes Based on Correlation Analysis Using ECO

The correlation analysis identifies the genes which have similar expression profiles, and those co-expressed genes may have similar function. In ECO, it provides correlation analysis for the query gene to all other genes in all cataloged EC groups, as well as in individual organs which have relatively large number of samples. This facilitates uncovering the function of novel or not much characterized genes based on correlation analysis.

**FIGURE 4:**
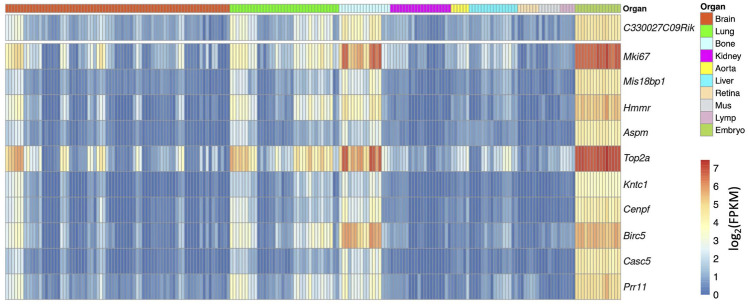
Heatmap overview of the top correlated genes to *C330027C09Ri*k. Each column shows one RNAseq sample, and its organ origin is colored on the top of the heatmap. The top 10 correlated genes are visualized. The heatmap color shows the expression level in each sample (log2 scaled FPKM) (high-resolution image is available in the ECO online database).

For example, we used a gene named *C330027C09Rik* as an example. *C330027C09Rik* did not yet have a clear gene name at the time of the gene assembly from the Ensembl database and was named after the full-length cDNA sequences from the RIKEN project (Hayashizaki, 2003). Among the top correlated genes, a list of well-known cell cycle-related genes appears, for example, *Mki67* and *Cenpf*, indicating that this gene maybe related with cell cycle (Figure 4). Interestingly, in the NCBI gene database, *C330027C09Rik* has been formally named as cell proliferation-regulating inhibitor of protein phosphatase 2A (*Cip2a*) (https://www.ncbi.nlm.nih.gov/gene/?term=C330027C09Rik). This confirmed the prediction from the correlation analysis.

## Discussion

Uncovering the EC transcriptional profile is critical to understand the EC functions in various vascular disease conditions. Previously, we have analyzed EC transcriptomes in normal mice brain (Vanlandewijck et al., 2018) and lung (He et al., 2018). It has improved the understanding of EC in these individual organs, while, on the public domain, many transcriptional profiling studies by different labs have accumulated extensive datasets for EC. However, using bioinformatics technologies to analyze these transcriptome data is a challenging task for many researchers. As such, it is of a great value to provide ECO, a user-friendly EC database, to explore expression profiles. Compared with the previously published EndoDB database (Khan et al., 2019), we have included all nine RNAseq studies in EndoDB, as well as eleven studies which were not presented there. ECO is a user-friendly web-based tool making the ever-increasing amount of EC transcriptome data easily accessible to non-bioinformatics researchers, as well as specialists as a resource of curated data. The core feature of ECO is one-click access to EC gene expression in different organs and alterations under different conditions for all genes on the genome. Unlike other databases, ECO dedicates to curate bulk RNAseq data from purified mouse EC under different conditions. All the data are processed using a standardized method for cross comparisons, and the results are visualized with easily understandable bar-plots. To make the users readily obtain the figures from ECO for presentation or publication usage, all the figures can be downloaded in the high-resolution PDF format.

ECO facilitates endothelial research with an integrated tool and resource for transcriptome analysis. With the friendly interactive interface, users can easily explore the published endothelial datasets from a variety of conditions, which may save some unnecessary animal experiments for vascular researchers. Also, ECO maximizes the value of published datasets by integrating them under a standardized platform. It may reveal potential global patterns which cannot be overserved from individual analysis. We expect that ECO will be a useful tool for researchers in the vascular community.

## Data Availability

The original contributions presented in the study are included in the article/Supplementary Material, further inquiries can be directed to the corresponding author.
